# Resin-based composite materials: elution and pollution

**DOI:** 10.1038/s41415-022-4241-7

**Published:** 2022-05-13

**Authors:** Steven Mulligan, Paul V. Hatton, Nicolas Martin

**Affiliations:** Academic Unit of Restorative Dentistry, School of Clinical Dentistry, The University of Sheffield, Sheffield, S10 2TA, UK

## Abstract

Pollution arises from all human activity and the provision of oral healthcare using resin-based composite restorative materials (RBCs) should be considered. This paper aims to provide a comprehensive review of the potential pollutant risk to the environment from the chemical compounds found in resin-based restorative materials, by including: 1) the principal pollutant compounds present in the resin matrix; 2) the degradation process of RBCs and its consequences; 3) the methods used for the detection and quantification of monomer elution and RBC microparticles; and 4) a review of the release mechanisms of eluates and RBC microparticles into the environment.

RBCs are pollutants by virtue of the compounds created during the degradation processes. These are in the form of the constituent eluted monomers and microparticles. Their impact on the environment and biodiversity is unknown. These materials are currently one of the main direct-placement restorative materials and their success is unquestionable when used and maintained correctly. Mitigation strategies for reducing the impact of pollution on the environment should be considered and implemented by all stakeholders and processes in the supply chain, from manufacturing, clinical use and waste management.

## Key points

- All the constituent components of resin-based composites have the potential to act as environmental pollutants as a consequence of their breakdown and subsequent elution.
- Microparticles have a pollution potential as they are easily dispersed in solution and have an increased surface area that potentiates the elution of monomers.
- Strategies to reduce their pollution impact should include: a) development of innovative direct-placement restorative materials; b) minimising waste; and c) providing good-quality preventive dentistry that minimises restoration failure and requirement for replacement.

## Introduction

Pollution can be defined as the presence in or introduction into the environment of a substance which has harmful or poisonous effects.^1^ Until recently, environmental pollution from the use of resin-based composite (RBC) materials has not been considered, with concern centred around biocompatibility issues such as cytotoxicity and oestrogenicity from the elution of constituent monomers.^2^^,^^3^The elution of monomers from RBC results in their release into the environment. Historically, environmental pollution has started with the release of a seemingly innocuous pollutant that over time builds to a point where a critical threshold is exceeded, causing unforeseen consequences. Within dentistry, much of the pollution discussion has focused upon amalgam by virtue of its mercury content; currently, the focus of the United Nations Minamata Convention Mercury Treaty of January 2013. This legally binding treaty has advised nations to phase down the use of dental amalgam on the basis of environmental pollution from its constituent mercury.^4^

Resin-based restorative materials, which are perceived to be either inert or have a reduced pollutant impact, are increasingly replacing the use of amalgam.^5^ This category of materials includes dentine adhesives, composites, resin-modified glass ionomers and resin-based luting agents, where most of which share a common organic polymer matrix (monomer before polymerisation) and a silane coupling agent. It is expected that RBC usage will increase in line with the mandates set by the Minamata Convention and the changes in treatment ethos.^6^^,^^7^

As per any manufactured item, all dental restorative materials have a potential pollutant effect on the environment. This will be associated with the manufacturing process, transportation, clinical use, disposal of waste material, human excretion and end-of-life of the person with the restorations. While there is some limited evidence of the harmful effect to health from constituents found in RBCs, such as bisphenol A (BPA) and methacrylate-based monomers, there is a lack of evidence that addresses the environmental pollutant potential of the plastic constituents of dental composite resin-based materials.^4^ Equally, there is no evidence of an environmental impact arising from this. It should be noted that the major environmental impacts from the use of these materials arises from the carbon footprint associated with manufacturing, distribution and disposal and use of auxiliary items (personal protective equipment for example), in addition to the plastic burden associated with packaging (designated as primary, secondary or tertiary, in accordance to its proximity to the material). Primary packaging acts as a container and delivery vehicle (compules, syringes); secondary packaging is often found in the form of polythene or aluminium/polyvinyl chloride laminate foils to protect the RBC from atmospheric humidity and light; and tertiary packaging takes the form of trays, boxes, cellophane and polythene wrapping. These all add a considerable environmental impact that should not be ignored but is outside the scope of this paper.^8^^,^^9^

The aim of this report is to provide a comprehensive review of the potential pollutant risk to the environment from the chemical compounds found in resin-based restorative materials, by considering: 1) the principal pollutant compounds present in the resin matrix; 2) the degradation process of RBCs and its consequences; 3) the methods used for the detection and quantification of monomer eluants and microparticles; and 4) a review of the release mechanisms of eluants and microparticles into the environment.

## Resin-based composite restoratives

RBC materials are used to restore the structural integrity, form and aesthetics of anterior and posterior teeth, enabling conservative cavity preparations on account of their adhesive properties.^10^ The range of applications of RBC extends to other disciplines of dentistry for use as a cement and as an indirect restorative material.^11^

RBC consists two phases: an inorganic filler dispersed within an organic methacrylate resin-based matrix phase. Other components key to controlling the polymerisation reaction include initiators, accelerators, inhibitors and photo-stabilisers (Table 1).

**Table 1:** Typical composition of representative RBC and dental adhesive^119^

Phase		Material
Resin matrix phase (typical monomers)		BisGMA UDMA TEGDMA HEMA*
Filler phase		Inorganic quartz and silica particles (silanated)
Other common constituents	Photoinitiator	Camphorquinone or proprietary*
	Accelerator ester	4-dimethylaminobenzoic acid ethyl*
	Inhibitor	3,5-di-tert-butyl-4-hydroxytoluene*
	Photo-stabiliser	2-hydroxy-4-methoxybenzophenone*
Key: * = Not universally used in all RBCs or substituted with related alternatives		

Common constituent monomers that form the matrix include bisphenol A-glycidyl methacrylate (BisGMA), urethane dimethacrylate (UDMA) and triethylene glycol dimethacrylate (TEGDMA). Various proprietary modified versions of these monomers also exist but are based around this set of methacrylate monomers. A brief description of each follows to aid with the understanding of the potential pollution mechanisms and pathways:

- BisGMA is the reaction product of bisphenol A and glycidyl ester methacrylate and contains pendant hydroxyl groups within its molecular backbone.^12^ In comparison to previously used RBC monomers, BisGMA exhibits reduced toxicity, shrinkage and volatility while maintaining a high modulus
- UDMA was developed as an alternative monomer as it has the advantages of reduced viscosity, increased filler loading and greater toughness when compared to BisGMA. UDMA is the product of 2,4,4-trimethylhexamethylene diisocyanate and 2-hydroxyethyl^13^
- Hydroxyethyl methacrylate (HEMA) is chemically synthesised from the reaction of methacrylic acid and ethylene oxide. HEMA is used in dental adhesives and is also used in photosensitive chemicals, adhesives, coating additives, thermosetting paints, sealants and personal care products. HEMA is also an intermediate in the production of other methacrylate esters^14^^,^^15^
- TEGDMA is a dimethacrylate monomer used mainly in dentistry; however, it is also used in industrial sealants, photopolymers, anaerobic adhesives, ultraviolent-cured coatings and fuel-resistant metal parts. TEGDMA is also an intermediate compound in the synthesis of other chemicals^16^
- BPA, while not a constituent of dental composites, is a degradation product of BisGMA and can be classed as a monomer of interest associated with RBCs and is recognised in the literature that it is present within dental composite.^17^

RBC filler particles are generally inorganic silica and quartz and range in size from nanometers to hundreds of micrometers, making up 45-75% of the composite volume.^18^^,^^19^ These particles are embedded within the resin matrix and are chemically united to the resin phase via a silane-coupling process.^20^^,^^21^^,^^22^ Filler particles are included to improve the physical properties of hardness, flexural strength, wear resistance, radiopacity and optical characteristics.^23^

RBCs are used either in a paste form as a direct-placement restorative material, or in a pre-polymerised state for machining in computer-aided design and computer-aided manufacturing (CAD/CAM) applications. RBC used as a direct-placement restorative is cured to a hard state via free-radical polymerisation during chemical or light activation or a combination of the two. Chemical activation requires the mixing of an activator such as benzoyl peroxide with an organic aromatic amine in a two-paste composite system. Light activation requires the use of a high intensity light of a blue wavelength (420-540 nanometres) and is more commonly used than chemical activated systems.^24^ Camphoroquinone is commonly used and is activated by blue light from a light-curing unit to start the polymerisation process. Alternative initiators include phenyl-propanedione (PPD), diphenyl (2,4,6-trimethylbenzoyl), phosphine oxide (TPO) and recently, ivocerine, a germanium-based initiator.^25^ These initiators require different activation wavelengths; PPD is below 350-490 nm and TPO is 380-425 nm.^26^

Stabilisers are used to help prolong the shelf life of RBC and prevent spontaneous polymerisation in ambient light when being used. Common stabilisers include monomethylhydroquinone, butylated hydroxytoludene and triphenyl antimony. The latter of these stabilisers has been identified as an eluate.^27^

Inorganic colour pigments allow RBC to have varying shades to allow matching to tooth colour. These inorganic pigments range from grey to red to yellow.

All of these chemicals described (and associated non-disclosed proprietary constituent organic and inorganic components) have the potential to be released as pollutants into the environment.

## Release of components from RBCs

A comprehensive review of the literature identified one key meta-analysis that provides a thorough review of the topic up to 2011 (van Landuyt *et al*., 2011) and a number of subsequent studies that satisfied the inclusion criteria for this review.^28^ Included studies considered the release of monomers and microparticles from resin-based composite restorative materials, the elution mechanisms, mechanisms of detection and the potential pathways to the environment.

Components from RBC can breakdown from the primary composite matrix and find their way into the environment. The pathway for this release is in the form of dissolved chemicals in solution (eluates) or particles, in the micron or nanoscale.

### Degree of conversion, elution and biodegradation processes

The direct placement of RBC restoratives and subsequent activation only achieves partial monomer conversion resulting in incomplete polymerisation. A maximum level of 60-75% monomer to polymer conversion is common^29^^,^^30^^,^^31^ and as low as around 30% at the base of a restoration.^32^ Conversely, 'factory' polymerised RBCs, typically used as blocs or ingots for machined CAD/CAM restorations, have a much higher degree of conversion.^33^ The mechanical properties of RBCs and therefore clinical durability and longevity are dependent on the degree of conversion of monomers to polymer. Thus, the concentration of released components from RBCs is dependent upon the degree of polymerisation with an inverse relationship between the leaching of resin components from RBC and monomer conversion.^34^Free or partly-linked monomers elute from the restorations and by extension, also from microparticulate waste.^32^ Therefore, incomplete polymerisation of direct-placement RBCs has the potential for leaching unpolymerised chemicals.^35^^,^^36^ The opposite is true; that the greater the degree of polymerisation of the material, the less elution of monomers occurs, with less potential biocompatibility or environmental pollution concerns. The ester bonds of common dental resin monomers, such as BisGMA, TEGDMA and UDMA, are susceptible to hydrolysis via host salivary hydrolases and esterases and cariogenic bacterial virulence upregulation, accelerating the biodegradation of RBCs.^37^ The resultant degradation of the resin matrix increases water sorption of the material, resulting in further hydrolysis, degradation and monomer release.

An *in situ* dental RBC restoration will consistently elute a small concentration of constituent monomers over a prolonged period of time; however, particulate RBC generated through milling, preparing, removing, finishing and polishing RBC has a more pronounced monomer release. The recognised elution of monomers from RBC over the short and longer-term, with further elution caused by bacterial degradation mechanisms, coupled with the large surface area of microparticulate waste, are contributory to increasing the pollution potential of RBC waste particulates.^38^^,^^39^ In summary, elution of the constituent monomers of RBC results from diffusion of unpolymerised monomers out of the material and also via hydrolytic or enzymatic degradation of the resin matrix.^40^

### What and how much is the release of components from RBCs?

RBC materials can find their way into the environment following chemical release (dissolution and elution of monomers), mechanical release (grinding into particles); or more commonly, a combination of the two degradation processes.

#### Elution of monomers from RBCs

The release of monomers from RBCs into solution is termed elution. Eluent refers to the solution or solvent used to extract the monomer (for example, acetonitrile in laboratory studies or saliva or water). Eluate is the combined extraction solvent (eluent) and the RBC monomers. When elution occurs, the chemical durability of the restoration is compromised, with subsequent biocompatibility and environmental pollution considerations. The dynamics of eluted monomers from RBC are the focus of current studies that seek to quantify this monomer release^28^^,^^41^^,^^42^^,^^43^ at different time points, both directly after placement^44^ and over longer periods of time.^38^^,^^45^^,^^46^

The elution of monomers from other RBC applications has also been investigated. These include intermediate restorative dental materials,^47^ dental cements,^48^ CAD/CAM blocks,^16^ endodontic sealers,^49^^,^^50^^,^^51^ bonding systems^52^ and occlusal splint materials,^53^ again with the focus on potential biocompatibility issues without consideration of environmental pollution.

##### Methods of quantification of monomers released from RBCs

Standard laboratory *in vitro* methods employed to extract, analyse and quantify RBC monomers include high performance liquid chromatography (HPLC),^54^ gas chromatography coupled to HPLC^42^ and solid phase micro-extraction (SPME).^55^ An effective methodology that meets these criteria utilises HPLC coupled with SPME.^46^

Methodological approaches can affect the nature of the results from a qualitative and quantitative perspective, that is, the type of monomers detected and their concentrations. To detect the monomers eluted from RBC, the chosen method needs to fulfil the following requirements: 1) be cost-effective, accurately quantitative and versatile enough to be used for a variety of solvents, namely urine, saliva, groundwater and landfill leachate; 2) should not alter the sample, 3) detect and quantify the eluted monomers in very low concentrations; and 4) reduce interferences or 'background noise' in complex environmental sample solutions.

Furthermore, the mechanisms and nature of elution is influenced by a number of factors that are detailed in Box 1. This elution process is further increased through the hydrolysis, photolysis and oxidation of the resin matrix and accelerated by microbial biodegradation.^45^^,^^56^^,^^57^^,^^58^^,^^59^^,^^60^Microorganisms capable of facilitating biodegradation of plastic materials can readily be isolated from the environment; therefore, disposed RBC will biodegrade and release monomers.^61^^,^^62^

The actual degradation of eluted monomers from RBC poses a significant analytical challenge. This degradation process results in further fragmentation of large molecular weight monomers to smaller compounds.^63^^,^^64^ An early study by Spahl *et al*. (1998) identified small amounts of BisGMA and UDMA but high concentrations of TEGDMA released from all RBC samples in water.^65^ Ortengren *et al*. (2001) investigated the water sorption and the elution of monomers from six different composite materials during water storage.^66^ Hydrolysis and oxidation of RBC in the presence of water has an important role in the degradation of RBC. Diffusion of water into the resin matrix of RBC promotes chemical degradation. TEGDMA was identified as the dominant monomer released from the RBC materials. UDMA and BisGMA release was noted from the RBC materials, albeit in much smaller concentrations. From elution kinetics studies, it is recognised that elution of the surface monomers occurs at approximately 100 times greater a rate than elution from the bulk of RBCs.^67^ Moharamzadeh *et al*. (2007) confirmed these findings in a study that investigated the release of the monomers BisGMA, UDMA and TEGDMA from three types of light-cured dental composite materials using HPLC.^54^ The study also identified TEGDMA in high concentrations in most but not all of the media samples. The high concentration of eluted TEGDMA found in these two studies is due to the increased relative hydrophilicity and lower molecular weight compared to BisGMA and UDMA, which impacts elution.

Box 1 Factors that influence the elution of monomers from resin-based dental composite restorations

- The composition of the monomer mixture and distribution of activators/inhibitors will affect polymerisation^120^
- The extent of the polymerisation reaction and double-bond conversion will impact the amount of unpolymerised monomer within the resin matrix.^121^ In light-polymerised RBC experiments, this is further influenced by light-curing unit factors, such as quality of light source, wavelengths of light omitted, distance to RBC and depth of light penetration^122^
- The solvent in which the experimental RBC resides impacts elution, with some organic solvents like ethanol or methanol resulting in greater elution rates than aqueous solvents like artificial saliva^28^
- The size and chemical nature, such as hydrophilicity of the monomers, would affect elution as, for example, a relatively large molecule such as BisGMA would not elute from the resin matrix as quickly as a small molecule such as TEGDMA
- The filler component influences elution, as the higher the load of filler materials within RBC, the volume of resin phase is reduced, with resultant reduced elution observed.^123^ The elution of monomers and oligomers from RBC impacts the biocompatibility and environmental impact of the material.

#### RBC microparticles

**Fig. 1:**
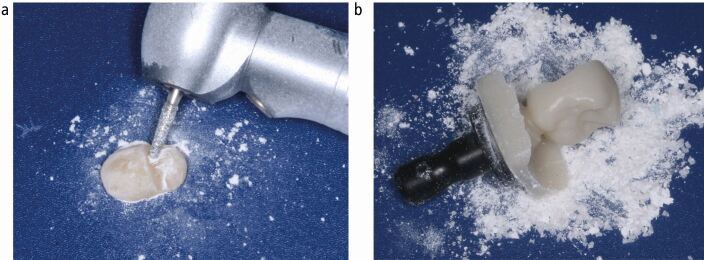
Microparticles generated from RBC material. a) One compule of direct-placement light-cured RBC. b) Full-coverage RBC crown ground from a CAD/CAM ingot

RBC microparticulate waste is generated at the chairside through the clinical grinding of *in situ* RBC restorations with high-speed rotary and abrasive burs/discs. This process commonly takes place during the removal of failed or aged RBC dental restorations and during the shaping, finishing and polishing of a directly placed restoration. Microparticles of RBC are also created from the subtractive milling and grinding of pre-polymerised RBC blocks to fabricate inlays, onlays, crowns, bridges and implant abutments (Fig. 1).

The effect of waste microparticles in the environment needs to be considered in terms of the actual nature and size of the microparticles and as a function of the release of monomers through elution and subsequent degradation processes. A review of freshwater microplastic pollution studies by Eerkas-Medrano *et al*. (2015), recommended a need for effective detection of microplastic particles and a better understanding of transport pathways, including wastewater.^68^ The analysis of elution from microparticulate RBC and its potential environmental impact is the focus of recent increased research attention.^69^^,^^70^^,^^71^^,^^72^^,^^73^

Beyond the potential pollutant effect of the actual microparticles, it is important to acknowledge the increased potential for elution of monomers from these particles. Free or partly-linked monomers elute from the resin matrix of direct-placement restorations and by extension, also from microparticulate waste.^32^^,^^74^

The recognised short- and long-term elution of monomers from RBC, the further elution caused by bacterial degradation mechanisms and the large surface area of microparticulate waste, are contributory to increasing the pollution potential of RBC waste particulates.^38^^,^^75^

##### Methods of quantification of microparticles released from RBCs

In order to consider the environmental impact of these particles, it is necessary to analyse the composition, size and behaviour. A range of techniques have been used for this purpose, of which, fourier transform infrared (FTIR) spectroscopy is the most widely used technique for the characterisation, identification and quantification of microplastics in wastewater samples.^76^^,^^77^ FTIR spectroscopy is a reliable, cost-efficient and relatively simple technique for the identification of microplastics. An additional advantage is that this method is non-destructive and FT-IR spectroscopy analyses have been successfully used for identifying microplastics in both sediment and water samples if the functional groups of the plastic have been pre-established.^78^^,^^79^ This method can be applied to samples from a dental origin in order to characterise microparticulates that are released from common dental applications into the environment, as the functional groups are known. Alternative techniques for analysis include Raman spectroscopy, sequential pyrolysis-gas chromatography coupled to mass spectrometry, infrared spectroscopy and combined FT-IR spectroscopy with microscopy.^80^^,^^81^^,^^82^^,^^83^

### Release mechanisms into the environment

A number of plausible release mechanisms are considered:^84^^,^^85^

- Disposal of RBC to landfill burial sites and incineration
- Human waste (saliva and urine) into wastewater and sewerage
- CAD/CAM milling and release of particulates into wastewater and sewerage
- Particulate release from clinical procedures (finishing and polishing or removal)
- Cremation of cadavers containing RBC into the atmospheric air
- Interment of cadavers containing RBC into groundwater.

#### Disposal of RBC to landfill and incineration

The majority of the waste produced in the dental industry is classified as municipal solid waste (MSW). MSW is a generic term that can also be applied to all residential, commercial and industrial waste.^86^ In the UK, MSW is recycled or sent to landfill; however, in many other countries, uncontrolled disposal of hazardous waste occurs, with potential for environmental harm.^87^ Adverse effects include leachate and gas emissions, fires and explosions, unpleasant odours, vegetation damage, ground water pollution, landfill settlement, climate change and air pollution; all concerns associated with landfill.^88^

RBC from dental surgeries that has expired its usage date and excess unused composite within discarded compules and syringes is considered as municipal solid waste and consequently disposed of in landfill sites. When disposed in this manner, landfill leachate can react with RBC allowing the release of its components. Landfill leachate is formed when precipitation percolates through the contents of a landfill site, promoting and assisting decomposition processes facilitated by bacteria and fungi. The temperature, pH and oxygen content of the landfill leachate solution change over time, affecting the reactivity of the solution. In a landfill site that receives a mixture of commercial, municipal and mixed industrial waste, a leachate composed of organic matter, inorganic ions and cations, heavy metal ions and xenobiotic compounds including persistent organic pollutants (POPs), will arise. This reactive leachate has the potential to allow breakdown and release of RBC into constituent components, including monomers, oligomers and BPA.^89^ Notwithstanding, the evidence supporting this release method is not clear. A laboratory microcosm study examining the reactiveness of RBC materials in landfill leachate concluded that the microbial activities and the increase of pH of this leachate environment may potentiate the release of TEGDMA, UDMA and BPA. However, these conditions do not affect the rate of release of Bis-GMA from dental composite materials.^90^ It should be considered that a breach of a landfill site through floods or coastal erosion has the potential to allow environmental pollution from RBCs.^91^

The potential of incineration as a suitable alternative to landfill has been investigated. A comparative study between the two methods concluded that bacteria-mediated degradation of RBC in landfill leachate with resultant increased release of BPA. Monomers are released from polymerised and unpolymerised RBC into landfill leachate over a prolonged period of time. Incineration of RBC results in the environmental release of significantly lower concentrations of monomers, elements and ions. Incineration is considered a viable alternative waste RBC disposal route, with a potentially lower environmental impact.^89^

#### Saliva and urine into wastewater

As highlighted earlier, during normal clinical use of RBC, elution of the constituent monomers and oligomers occurs, as complete polymerisation is not possible.^29^^,^^30^^,^^31^ Thus, unpolymerised RBC components are excreted in human waste after placement into the environment. A key large-scale study by Kingman *et al*. (2012) provides a very valuable insight into this pollution stream, identifying that monomer eluates (BisGMA, TEGDMA and BPA) found in urine and saliva can be released into the environment up to 30 hours after the placement of a RBC restoration. The application of dental dam reduced the quantities of monomers detected in saliva significantly (by four for BisGMA).^92^

#### Microparticulates and microplastics

RBC microparticulates are distinct from microplastics, as they are a heterogenous combination of polymer and glass filler, whereas microplastics are generally homogenous and made of one polymer, such as polypropylene. Microplastics are defined as plastic particles smaller than 5 mm and represent an increasing proportion of plastic debris released into the environment.^93^ Microplastics act as direct pollutants and can attract and bind to biotoxins known as POPs, such as polychlorinated biphenyl (PCB).^94^ There is speculation that adsorption of POPs to microplastics increases the possibility of access to the food chain via the process of bioaccumulation.^95^ Ingestion of microplastics has been documented in plankton, barnacles, mussels, fish and seabirds.^96^ Microplastic particles are found in many species of North Sea fish, including popular edible species such as haddock, cod and herring.^97^ RBC microparticulates are reactive (they elute the monomer constituents) and are charged and can therefore potentially attract and bind other compounds in the same manner as microplastics can.^98^ Methods of detection and quantification of microplastics are improving to help better understand this phenomenon.^99^

##### Microparticles from routine dental treatment

The clinical process of polishing, replacement or adjustment of a RBC restoration generates particulate waste.^100^ This waste material is removed from the oral cavity by the use of an aspirator and is disposed to wastewater, which proceeds to the environment via municipal sewerage. The size of these particulates ranges from nanoscale to around 10 μm.

The CAD/CAM subtractive (removal of material) manufacturing process of grinding pre-polymerised RBC ingots generates significant volumes of microparticulate powder that is often disposed into landfill or via wastewater discharge into municipal sewerage.

Beyond the actual impact of the microparticles, the actual pollutant potential associated with monomeric elution from these microparticles is unknown. The pollutant potential is determined by the monomeric composition, the age of the restoration (and therefore how much previous elution of monomer has occurred), the size and surface area of the microparticles released and the extent of polymerisation (which can be influenced by operator factors and material chemistry). In this context, it is important to note that the breakdown and potential elution of monomers from the two processes (clinical and CAD/CAM grinding) is likely to differ on account that the CAD/CAM blocks are highly polymerised RBC, compared to the direct-placement materials, with a range of 50-70% conversion rate.^29^^,^^30^^,^^31^

The characterisation of microparticles arising from either grinding direct placement restorations or CAD/CAM RBC ingots after 12 months ageing in water has been investigated.^85^^,^^98^ The direct-placement commercial RBC microparticles were clearly discernible after this time, with consistent alteration of the outermost surfaces of particles, suggesting particle breakdown and monomer elution. The previously reported study by Polydorou (2020) evaluated the release of BPA in wastewater after grinding of resin composites and tested three filtration materials.^70^ BPA was detected in all solutions of ground microparticulate commercial resin-based composites, highlighting that BPA can be released in wastewater during dental procedures. The charged nature of microparticulate RBC and experiments involving catalytic carbon filtration have been suggested for RBC microparticulate and BPA reduction in wastewater when considering remediation strategies.^70^^,^^71^^,^^72^^,^^85^

#### Pollution after end of life

Interment and cremation are the most common approaches to the management of human remains, which include a high volume of dental restorations and prostheses. Given the high number of resin-based composite restorations, this potential environmental pollution pathway also merits attention.

As life expectancy increases in line with the number of dentate patients containing restorations, future repair and replacement of restorations with RBC means the amount of RBC that will be placed in patients for future release into the environment will increase.

##### Interment

The environmental impact associated with the burial of cadavers containing RBC has the potential for the release of eluates into the environment via percolation of groundwater. Understanding the extent and rate of elution of materials into groundwater is complicated by material-based factors, such as: the type of dental RBC and its composition at burial; how long it has been *in situ*; and treatment-dependent factors, such as how well polymerised the material was. This is then further compounded by interment site and method-related factors:^101^

- Geological and hydrogeological characteristics of the soil, including soil type, permeability and porosity, pH and ability of groundwater to diffuse, would impact the release of eluted monomers
- Microbiological characteristics of the soil and groundwater^89^
- Mechanical, structural and resistance parameters of the soil
- Coffin or other container construction used. The less permeable or biodegradable, the less release into groundwater
- Land cover and topography will affect infiltration and water permeation
- Climate: degradation of RBC and elution rate are temperature dependent with positive correlation between increased temperature and elution of monomers
- Depth of the unsaturated zone of the soil has an impact because as well as acting as a barrier to contamination of an underlying aquifer, this can also present a means for infiltration of oxygen that may aid decomposition and the elution process.

An *in vitro* investigation into the elution of monomers from RBCs into groundwater identified that low concentrations of monomers are released into groundwater over a prolonged time from RBC.^102^ It is clear from these statistics and the highlighted trend of increasingly higher RBC use in the future, that understanding the consequences of elution of monomers from RBC into the environment from cadavers requires further investigation.

##### Cremation

Cremation is the process of combustion, vaporisation and oxidation of human remains. In Europe, over 150,000 cremations occur each year in the 1,000+ crematories in operation.^103^ Temperatures of 800 °C or higher are required over a time period of between 1-2 hours. During the cremation process of human cadavers, a number of emissions are released into the environment.^104^ These pollutants include mercury compounds (principally from dental amalgam), dioxins, furans, hydrogen chloride, nitrogen oxides, carbon monoxide and volatile organic compounds. Through the use of established combustion methods, secondary combustion chambers and filters, the majority of pollutants released can be maintained below regulation limits. In addition, concentrations of mercury, hydrogen chloride, dioxins and furans can be monitored in additional arrestment chambers.^105^

There is no published data that characterises the pollution potential from this pathway.

## Discussion

The oral healthcare community recognises that we have a joint responsibility to deliver products and interventions that improves oral health and does so in an environmentally sustainable manner.

In the last decades, the combined efforts of the oral health industry, researchers, governing bodies and the oral healthcare profession have been hugely successful in the delivery of a sophisticated understanding and knowledge of oral and dental diseases, treatment strategies and modalities. This includes the innovation and development of excellent technologies, materials and products to provide this care, including state-of-the-art RBC restoratives. These combined efforts have, to date, been largely focused on the prevention and management of oral diseases. Today, we have a further understanding of the need to ensure that optimal oral healthcare provision should also minimise unnecessary CO_2_e emissions and environmental pollution as much as possible. In the future, with further understanding, it is hoped that oral healthcare provision becomes carbon-neutral and pollution-free.

This article has summarised the literature regarding the release of monomers from RBC and RBC microparticles, with a focus on environmental pollution. It identifies that all the constituent components of RBCs have the potential to act as pollutants as a consequence of their breakdown. This may be in the form of eluates, microparticles, or a combination of the two. The breakdown of these materials can occur through a range of different pathways.

It is important to highlight the distinction between pollution and the impact of this on the environment and biodiversity. While we recognise, from *in vitro* studies, the pollution potential of these materials, we do not have, to date, any evidence that these materials have an adverse impact on the environment and biodiversity.

Characterisation of the pollution potential of these materials is very challenging and limited to *in vitro* laboratory studies. The reasons for this are associated with the nature of the chemicals, their combination with other environmental substances when eluted (organic and inorganic) to form complex substances, the limitations of the analytical techniques and the interpretation of the results. Notwithstanding, the evidence to date is conclusive in that elution of monomers arising from RBCs does occur and that these are released into the environment. TEGDMA is the most dominant monomer released from RBCs but this is a function of its relatively high hydrophilicity and its lower molecular weight. It is pertinent to note that components released from RBCs into the mouth may react with other substances (eg saliva, food bolus and gastric contents) and may be inactivated in the process. As such, it is difficult to study and fully understand the impact of specific constituents of RBCs.

Microparticles have a pollution potential associated with their small size that makes them easily dispersed in solution and their increased surface area that potentiates the elution of monomers. Microparticles also have a tendency to create agglomerates with other substances, creating potentially more complex polluting substances. Beyond the actual impact of the microparticles, the actual pollutant potential associated with monomeric elution from these microparticles is unknown.

A number of plausible pollution pathways are considered for RBCs, for which we have very limited knowledge. Of these, the release of microparticles and eluates during the clinical removal, finishing/polishing and CAD/CAM milling of restorations and their subsequent discharge into wastewater and landfill burial have received the greatest research attention. The relevance of these processes in generating microparticles becomes evident if we consider the number of RBC restorations that are placed and replaced/refurbished worldwide. It is estimated that in 2015, 800 million direct RBC restorations were placed; a figure based on industry sales figures.^106^ Estimates of ten-year failure rates for RBCs (restorations that require removal and replacement or refurbishment) range between 32 million posterior restorations (Heintze *et al*. 2012) and 48 million of all restorations (Beck *et al*. 2015).^107^^,^^108^ Accepting an average figure of 40 million and considering the average weight of a RBC restoration to be 0.3 g, it is possible to calculate the approximate mass of particulate waste generated and released into municipal wastewater to be in the range of 12 tonnes per year. While this may be considered to be a relatively small amount of pollution compared to other industries; the oral healthcare industry has an equal level of responsibility to manage and minimise its pollution impact, regardless of the magnitude of this.

With the impending demise of dental amalgam, RBCs are the only alternative direct-placement restorative materials with universal application in all the dentition. Strategies to reduce their pollution impact should be:^109^^,^^110^

1. To promote research and development to create innovative direct-placement materials that have low technique sensitivity and are cariostatic, easily placed, durable and have a low/zero pollution impact^111^^,^^112^
2. To adapt our processes throughout the supply chain to minimise the pollutant impact from these materials by minimising waste at all stages, through careful attention to the manufacturing processes, distribution, clinical use and associated logistics
3. To suggest clinicians should engage and promote effective caries prevention regimes that will reduce the failure rate of RBCs and their need for replacement.^113^ Ensure that procedures are executed and maintained to the very highest standards to avoid restoration removal and replacement.^114^ This requires the use of high-quality materials that are placed following evidence-based protocols. The repair and replacement of restorations leads to premature loss of teeth through the process known as the restorative cycle.^115^^,^^116^ Slowing down or stopping the restorative cycle will have a dual environmental positive effect:
  Reduced use of materials, creating less manufacturing waste, less packaging and less clinical waste
  Reduced number of patient visits for routine dental care that equates to reduced travel and thus a reduced patient-based carbon footprint.

Elution of monomers can be mitigated through the use of clinical techniques and effective light curing to: maximise polymerisation conversion rates; undertake the replacement of RBC with dental dam isolation to reduce the concentration of monomer elution in saliva and; use glycerine gel on the final light-cured surface of RBC to avoid the oxygen-inhibited layer and in this way, limit the amount of unpolymerised monomer that is present and removed during finishing/polishing of the restoration :^117^

- Further clinical strategies to reduce the generation, release and impact of microparticles are: Modify treatments by not overbuilding restorations and therefore requiring more instrumentation
- Consider repairing restorations rather than replacing when clinically appropriate
- Dispose of CAD/CAM waste responsibly and not down the sink
- Development of RBC materials with improved degrees of monomer conversion
- Reduce the restorative need through prevention
- Development of adjunctive technology to capture microparticulate waste at the point of generation (clinic or laboratory).

This review has identified the need for a better understanding of these pollution pathways to aid the development and implementation of restorative material technology, clinical practice protocols, technologies to mitigate the pollution impact and associated legislation and policies that support these strategies.

## Conclusions

RBC is currently the most suitable direct aesthetic restorative dental material and its clinical success is unquestionable when used and maintained correctly. This review has identified that resin-based composites may release monomers and microparticles that are potential pollutants. We do not have, to date, any evidence that these materials have an adverse impact on the environment and biodiversity. However, these substances certainly have the potential to do harm, especially if critical thresholds are exceeded, indicating more research is required. Mitigation strategies for reducing the impact of pollution on the environment should therefore be considered and implemented by all stakeholders, from manufacturers, through clinical use or disposal and on to waste management. Recommended approaches include careful supply chain management to avoid accumulation of products beyond their use by date and delivery of high-quality clinical services to ensure that restorations deliver the greatest longevities possible. Prevention and management of oral diseases through good-quality care and treatment is key to improving sustainability by reducing the need for dental materials and associated negative environmental impacts.^118^
